# A comprehensive analysis of age-related metabolomics and transcriptomics reveals metabolic alterations in rat bone marrow mesenchymal stem cells

**DOI:** 10.18632/aging.203857

**Published:** 2022-01-30

**Authors:** Xiao Yu, Hui Sun, Xingyu Gao, Chang Zhang, Yanan Sun, Huan Wang, Haiying Zhang, Yingai Shi, Xu He

**Affiliations:** 1 The Key Laboratory of Pathobiology, Ministry of Education, College of Basic Medical Sciences, Jilin University, Changchun, Jilin 130021, China

**Keywords:** BMSCs, aging, metabolomics, transcriptomics, lipid metabolism

## Abstract

The functions of stem cells decline progressively with aging, and some metabolic changes occur during the process. However, the molecular mechanisms of stem cell aging remain unclear. In this study, the combined application of metabolomics and transcriptomics technologies can effectively describe the possible molecular mechanisms of rat bone marrow mesenchymal stem cell (BMSC) senescence. Metabolomic profiles revealed 23 differential metabolites which were abundant in “glycerophospholipid metabolism”, “linoleic acid metabolism” and “biosynthesis of unsaturated fatty acids”. In addition, transcriptomics analysis identified 590 genes with enormously differential expressions in young and old BMSCs. KEGG enrichment analyses showed that metabolism-related pathways in BMSC senescence had stronger responses. Furthermore, the integrated analysis of the interactions between the differentially expressed genes (DEGs) and metabolites indicated the differential genes related to lipid metabolism of Scd, Scd2, Dgat2, Fads2, Lpin1, Gpat3, Acaa2, Lpcat3, Pcyt2 and Pla2g4a may be closely associated with the aging of BMSCs. Finally, Scd2 was identified as the most significant DEG, and Scd2 over-expression could alleviate cellular senescence in aged BMSCs. In conclusion, this work provides a validated understanding that the DEGs and metabolites related to lipid metabolism present more apparent changes in the senescence of rat BMSCs.

## INTRODUCTION

Along with social progress and rapid development of economy, a graying society faces inevitable changes, and aging is one of the most important reasons for its occurrence [1]. Aging is an unavoidable physiological process that caused by senile tissues and cells and declined organ functions [2]. The complexity of aging is partly reflected in the diversity of its hallmarks, which can be condensed into the following three categories: primary, or causes of age-associated damage; counteractive, or responses to the damage; and integrative, or consequences of the responses and culprits of the aging phenotypes [3, 4]. Based on the “theory of stem cell aging”, adult stem cells are primarily in charge of age-related loss of cellular functions and aging [5]. A large number of studies have shown that aging is one of the greatest risk factors associated with an array of morbidities including diabetes, cardiovascular diseases, musculoskeletal, neurodegenerative conditions and various malignancies [6]. Therefore, in-depth exploration on activating senescent stem cells is the basic and bottleneck for delaying individual aging and preventing age-related diseases.

Aging is closely related to disturbance in the maintenance of metabolism. With cellular senescence, a large number of neutral amino acids such as valine, isoleucine and glycine may be used as alternative energy sources to maintain energy homeostasis [7]. Some studies reported that the anti-aging mechanisms of *C. elegans* and rats may be related to the regulation of lipid metabolism [8, 9], and lipids can modulate the biological characteristics of stem cells by influencing energy storage, plasma membrane composition, signal transduction and gene expression changes [10]. In recent years, a study revealed the important role of age-related lipid metabolism in abnormal differentiation of BMSCs by integrating lipidomics and transcriptomics [11]. Therefore, it is vital to understand the aging process and make a comprehensive and systematic study on metabolic pathways. Our understanding of metabolic networks and biological systems can be greatly boosted by integrating metabolomics and transcriptomics [12].

The concept of metabolomics was proposed by Nicholson et al. in 1999, and it is an omics technology that developed rapidly in the 1990s after genomics, transcriptomics and proteomics research [13]. It is a discipline in which qualitative and quantitative detection of all low molecular mass metabolites of a certain organism or cell is carried out to analyze the alterations of metabolite spectrum in living cells [14]. Lawton et al. found that extraordinary changes in relative concentrations of more than 100 metabolites were associated with aging, and Yi et al. demonstrated that the differential metabolites in human umbilical vein endothelial cells (HUVECs) from passage 3 to 18 were principally involved in 14 significantly altered metabolic pathways [15]. Although the mechanisms of aging cannot be fully revealed through the use of metabolomics, it is a promising tool in aging research [16]. Transcriptomics is a high-throughput technology that can measure the whole genome and identify new candidate pathways and targets. It has been successfully applied to identify the pathways and networks that control the complicated biological process of aging [17]. In recent years, there have been some reports on the metabolic regulation of MSC aging, but the specific mechanism has not been fully elucidated.

BMSCs have the merits of low immunogenicity, strong vitality, uniform biological characteristics and no ethical controversy [18]. Therefore, the metabolomics and transcriptomics of BMSCs derived from young and aged rats were conducted to shed light on the underlying molecular mechanisms in stem cell senescence. Then the potential biomarkers and related genes in old BMSCs could be screened by using high-throughput technologies and powerful bioinformatics.

## RESULTS

### Multivariate statistical analysis

Based on the data of non-targeted metabolomics, we established a multivariate statistical analysis model to reveal the changes of metabolites in old BMSCs and young BMSCs. Intensities were corrected for signal drift and batch effect by fitting a locally quadratic (loess) regression model to the median intensity of pooled QC samples. After correction, the median area of all pooled QC samples was now the same. Metabolites with a coefficient of variation (CV) in QC samples >25% were then filtered out, due to their unstable quantifiability. In the end, a total of 1886 annotated metabolites were obtained, including positive ion modes and negative ion modes, as well as some details information such as m/z, retention time and peak intensity.

**Figure 1:**
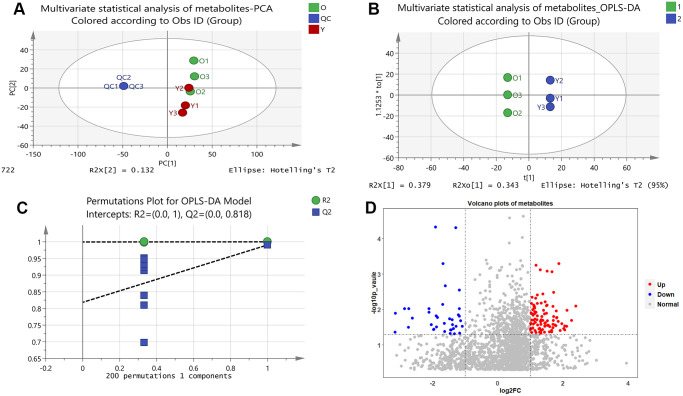
**Results of multivariate statistical analysis of metabolomics.** (**A**) Multivariate statistical analysis of metabolites-PCA. PC1 and PC2 represent the degree of interpretation of the model with the first and second principal component ranking in the principal component analysis. (**B**) Multivariate statistical analysis of metabolites-OPLS-DA. The separation of the two classes of observations occurs in the horizontal (t1) direction; The vertical (t1o) direction indicates intra-class variability. (**C**) Permutations Plot for OPLS-DA Model. For the selected Y variable, the figure shows the R2 and Q2 values of the original model (far right) on the vertical axis; The horizontal axis shows the correlation between the substituted Y vector and the original Y vector of the selected Y. (**D**) Volcano plots of metabolites. The red dots represent up-regulated metabolites and the blue dots represent down-regulated metabolites, and the grey dots represent that the metabolites were not significantly changed.

Unsupervised principal component analysis (PCA) of the metabolic data was used to observe grouping trends, prominent outliers, and clustering between display groups to provide an initial assessment of metabolic disturbances. PCA score plots showed the distribution between the old BMSCs and young BMSCs as well as QC samples in two dimensions (Figure 1A). According to Supplementary Figure 1, the QC samples were tightly clustered within 95% confidence interval, which proved that the method was steady and the instrument had good repeatability.

We further developed a supervised OPLS-DA methodological model (R2X = 0.946, R2Y = 1, Q2 = 0.991) to better account for changes in metabolites between the old and young cells (Figure 1B). A permutation test of 200 random numbers was used to verify the OPLS-DA model and the result showed that all blue Q2-values to the left were lower than the original points to the right (Figure 1C), which proved the OPLS-DA model was effective and stable. Judging by the OPLS-DA model, there was a distinct difference between the two groups, indicating an apparent disturbance in the senescent BMSCs.

Volcano plots were constructed to determine the difference in metabolites between the old and young cell groups (Figure 1D). In the diagram, each dot represents a metabolite. As shown in the Figure 1D, a total of 130 metabolites showed visible alterations, 92 metabolites were up-regulated (red dots), 38 metabolites were down-regulated (blue dots), and most of the other metabolites were not significantly changed (grey dots). In the volcanic plots, the further away a point was from the origin, the more it contributes to the distinction between the two groups. Thus, the distant points from the origin were candidate biomarkers for senescent BMSCs.

### Identification of candidate biomarkers

**Figure 2:**
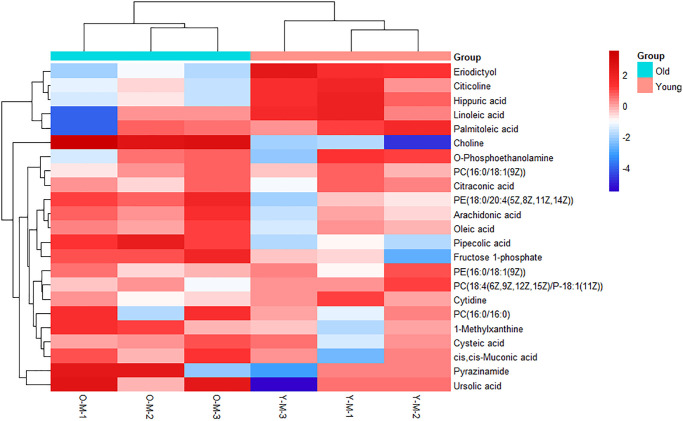
**Heatmap of metabolites with significant differences.** Each row represents a sample and each column represents a metabolite. Red is the high expression level, blue is the low expression level.

On the basis of the accuracy of MS data, the exact mass of each feature was submitted to ChemSpider with 4 databases selected (BioCyc; Human Metabolome Database; KEGG; LipidMAPS). According to the predicted value of VIP in the OPLS-DA model and the return *p*-value of the *t*-test, 23 metabolites with significant changes were screened as candidate biomarkers (VIP > 1 and *p*-value <0.05), which were displayed using a heatmap (Figure 2). Details of 23 differential metabolites are shown in Table 1. As shown in the heatmap, the differential biomarkers can distinguish the old BMSCs group from the young group, indicating that the biomarkers we have obtained were reliable.

**Table 1:** Identified metabolites in old BMSCs compared with young BMSCs.

**mz/rt**	**Hit name**	**HMDB**	**KEGG**	***P*_value**	**vip_value**
758.6286/1.7	Choline	HMDB0000097	C00114	0.006608	1.45253
759.5756/0.8	PC(16:0/18:1(9Z))	HMDB0007972	C00157	0.043813	1.45682
733.5621/1.2	PC(16:0/16:0)	HMDB0000564	C00157	9.3E-05	1.35649
763.5515/1.1	PC(18:4(6Z,9Z,12Z,15Z)/P-18:1(11Z))	HMDB0008260	C00157	0.014569	1.47597
751.5369/1.1	Arachidonic acid	HMDB0001043	C00219	0.008712	1.41671
757.5616/0.8	Citicoline	HMDB0001413	C00307	0.045412	1.44704
763.5146/1.2	O-Phosphoethanolamine	HMDB0000224	C00346	0.015577	1.46811
717.5311/0.8	PE(16:0/18:1(9Z))	HMDB0008927	C00350	0.009709	1.32245
767.5459/1.1	PE(18:0/20:4(5Z,8Z,11Z,14Z))	HMDB0009003	C00350	0.043893	1.4927
607.3775/1.3	Pipecolic acid	HMDB0000070	C00408	0.000414	1.12903
585.363/2.6	Cytidine	HMDB0000089	C00475	0.001039	1.09283
842.5101/13.5	Cysteic acid	HMDB0002757	C00506	0.007164	1.95638
817.5381/1.3	Oleic acid	HMDB0000207	C00712	0.009268	1.83144
736.5279/1.8	Fructose 1-phosphate	HMDB0001076	C01094	0.019808	1.36175
97.9673/19.7	Hippuric acid	HMDB0000714	C01586	4.93E-05	2.68782
555.3523/0.9	Linoleic acid	HMDB0000673	C01595	0.00279	1.01919
95.9148/10.8	Pyrazinamide	HMDB0014483	C01956	0.002584	2.52509
96.0317/2.4	Citraconic acid	HMDB0000634	C02226	0.005057	2.54612
97.9753/1.3	cis,cis-Muconic acid	HMDB0006331	C02480	0.00629	2.73208
90.031/2.6	Eriodictyol	HMDB0005810	C05631	0.029214	2.37657
767.5227/1.2	Palmitoleic acid	HMDB0003229	C08362	0.003539	1.48757
626.5274/1.8	Ursolic acid	HMDB0002395	C08988	0.016756	1.17649
849.2433/0.7	1-Methylxanthine	HMDB0010738	C16358	0.030485	2.01903

### Metabolic pathway analysis

**Figure 3:**
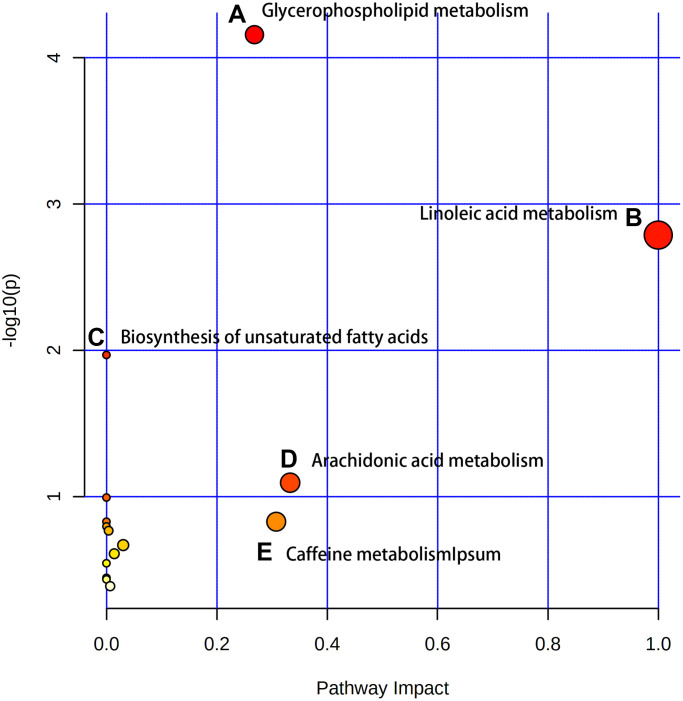
**The enriched pathway analysis of metabolites.** There are five metabolic pathways with significant changes: (**A**) Glycerophospholipid metabolism; (**B**) Linoleic acid metabolism; (**C**) Biosynthesis of unsaturated fatty acids; (**D**) Arachidonic acid metabolism; (**E**) Caffeine metabolism.

After identifying the candidate biomarkers, we enriched 15 disordered metabolic pathways by using MetaboAnalyst 4.0: glycerophospholipid metabolism, linoleic acid metabolism, biosynthesis of unsaturated fatty acids, arachidonic acid metabolism, caffeine metabolism, taurine and hypotaurine metabolism, phenylalanine metabolism, alpha-linolenic acid metabolism, glycosylphosphatidylinositol (GPI)-anchor biosynthesis, fructose and mannose metabolism, sphingolipid metabolism, lysine degradation, cysteine and methionine metabolism, glycine, serine and threonine metabolism and pyrimidine metabolism (Figure 3). Details of these disordered metabolic pathways are shown in Table 2.

**Table 2:** Disordered metabolic pathways.

**Pathway name**	**Total**	**Hits**	***p* value**	**−log10 (*p* value)**	**Impact**
Glycerophospholipid metabolism	36	5	6.96E-05	4.1575	0.26825
Linoleic acid metabolism	5	2	0.00163	2.7877	1
Biosynthesis of unsaturated fatty acids	36	3	0.01076	1.9682	0
Arachidonic acid metabolism	36	2	0.080362	1.095	0.33292
Caffeine metabolism	12	1	0.14845	0.82841	0.30769
Taurine and hypotaurine metabolism	8	1	0.10146	0.99368	0
Phenylalanine metabolism	12	1	0.14845	0.82841	0
Alpha-Linolenic acid metabolism	13	1	0.15983	0.79634	0
Glycosylphosphatidylinositol (GPI)-anchor biosynthesis	14	1	0.17106	0.76685	0.00399
Fructose and mannose metabolism	18	1	0.21458	0.66841	0.03037
Sphingolipid metabolism	21	1	0.24578	0.60945	0.0142
Lysine degradation	25	1	0.28556	0.5443	0
Cysteine and methionine metabolism	33	1	0.35922	0.44464	0
Glycine, serine and threonine metabolism	34	1	0.3679	0.43427	0
Pyrimidine metabolism	39	1	0.40967	0.38757	0.0068

### Transcriptomics profiling of the BMSCs

**Figure 4:**
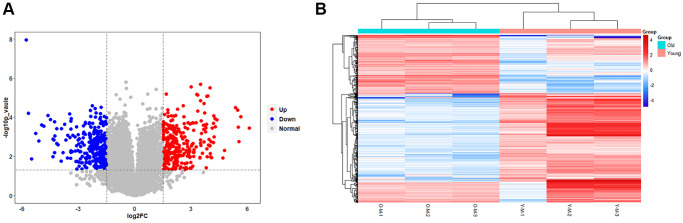
**Results of statistical analysis of transcriptome differences.** (**A**) Volcano plots of mRNAs. The volcanic map is drawn with -log10 (*p*-value) as the vertical axis and log2 (Fold Change) as the horizontal axis, which shows the changes of mRNA expression in the old BMSC group and the young BMSC group. The red dots represent up-regulated mRNAs and the blue dots represent down-regulated mRNAs, and the grey dots represent that the mRNAs were not significantly changed. (**B**) Heatmap of DEGs. Each row represents a sample and each column represents a mRNA. Red is the high expression level, blue is the low expression level.

The results of the transcriptional group showed that there was a remarkable difference in gene transcription levels between the old BMSC group and the young BMSC group. A total of 16205 RNAs were detected, and 14574 meaningful RNAs (FPKM > 0.5) were screened. We further confirmed the screening conditions of |log2(foldchange)|> 1.5 and *p*-value < 0.05. Based on this condition, 590 RNAs with significant differences were considered to be DEGs. Compared with the young group, the expression of 311 RNAs were down-regulated and 279 RNAs were up-regulated in the old BMSCs group. The volcanic plots visually showed the variation trend of these RNAs (Figure 4A). We proved further that the 590 DEGs identified in the form of a heatmap (Figure 4B), and we found that DEGs could separate the old BMSC group from the young group, indicating that the DEGs we obtained were reliable.

### GO and KEGG enrichment analyses of DEGs

**Figure 5:**
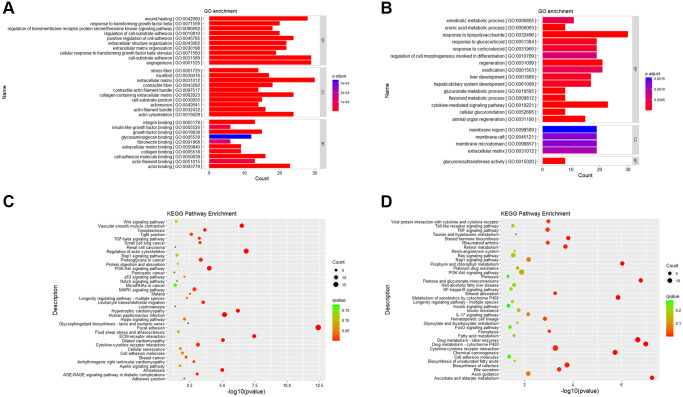
**Gene ontology and KEGG pathway enrichment results.** (**A**) GO enrichment analysis of up-regulated DEGs. (**B**) GO enrichment analysis of down-regulated DEGs. (**C**) KEGG enrichment analysis of up-regulated DEGs. (**D**) KEGG enrichment analysis of down-regulated DEGs. Abbreviations: BP: Biological Process; CC: Cellular Component; MF: Molecular Function.

GO and KEGG enrichment analysis are based on different angles to better explain and understand the biological significance of DEGs. GO enrichment analysis focuses on the functional enrichment of DEGs. From the results of GO enrichment analysis, we found that up-regulated DEGs were enriched in 270 significant items (Supplementary Table 1), and down-regulated DEGs were enriched in 73 significant entries (Supplementary Table 2). Among the GO items into which up-regulated DEGs were enriched, we selected the top 10 entries of BP (biological process), CC (cellular component) and MF (Molecular Function) to draw bar charts (Figure 5A). Among the GO entries enriched by down-regulated DEGs, top 15 of BP, all CC and all MF entries were drawn as bar charts (Figure 5B).

KEGG enrichment analysis focuses on gene pathway analysis. From the results of pathway enrichment, we found that 279 up-regulated genes enriched in 170 KEGG pathways (Supplementary Table 3) and 311 down-regulated genes enriched in 240 KEGG pathways (Supplementary Table 4). It was enriched in 38 significantly up-regulated pathways (Figure 5C) and 38 down-regulated pathways (Figure 5D).

### Protein-protein interaction network

**Figure 6:**
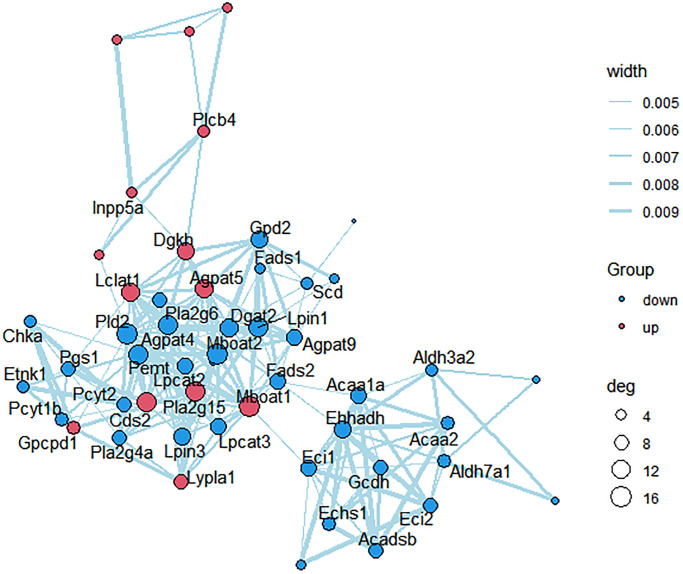
**PPI network construction.** Each node represents one differentially expressed protein. Each edge represents regulation. Red, up-regulated expressed protein; blue, down-regulated expressed protein.

KEGG pathways related to lipid metabolism were selected, and protein-protein interaction (PPI) network was further constructed between the des enriched in these pathways (Figure 6). Scd, Mboat2, Agpat5, Dgat2, Fads2, Lpin1, Acaa2, Lpcat3, Pcyt2 and Pla2g4a were strongly correlated with other proteins (connected with >3 proteins).

### Integrative transcriptomic and metabolomic molecular profiling analysis

**Figure 7:**
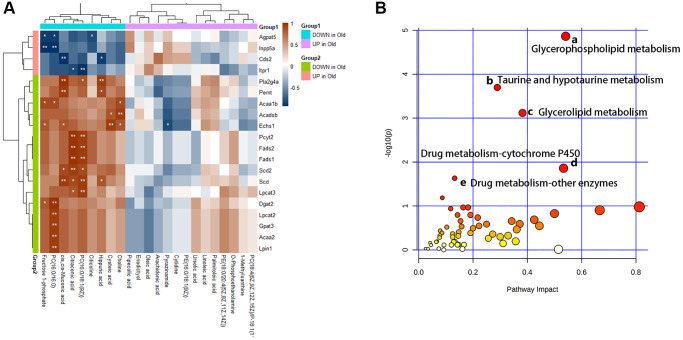
**Multiple omics combined analysis results.** (**A**) The heatmap of differential metabolites and genes. (**B**) The enriched pathway analysis of integrative data. There were five metabolic pathways with significant changes: (**a**) Glycerophospholipid metabolism; (**b**) Taurine and hypotaurine metabolism; (**c**) Glycerolipid metabolism; (**d**) Drug metabolism - cytochrome P450; (**e**) Drug metabolism - other enzymes.

Furthermore, we analyze the changes of metabolic pathways in the process of cellular senescence by integrating metabolomic and transcriptome data, so as to better understand the mechanisms of BMSCs senescence from a metabolic point of view. 52 KEGG metabolic pathways (Figure 7A) were enriched by integrating data, of which 5 metabolic pathways (*p*-value < 0.05 and Impact > 0.1) had visible changes in the process of cellular senescence, and 29 metabolic pathways (*p*-value > 0.05 but Impact > 0.1) were potentially associated with cellular senescence. In order to understand the metabolic response of senescent BMSCs at the transcriptome level, correlation analysis heat map of the connection between differential metabolites and DEG was established (Figure 7B). The DEGs involved in lipid metabolism exhibited the most dramatic changes in BMSC senescence. The differential metabolites in lipid metabolism were extremely correlated and statistically significant with DEGS.

### Age-associated changes in BMSCs and validation of mRNA expression

**Figure 8:**
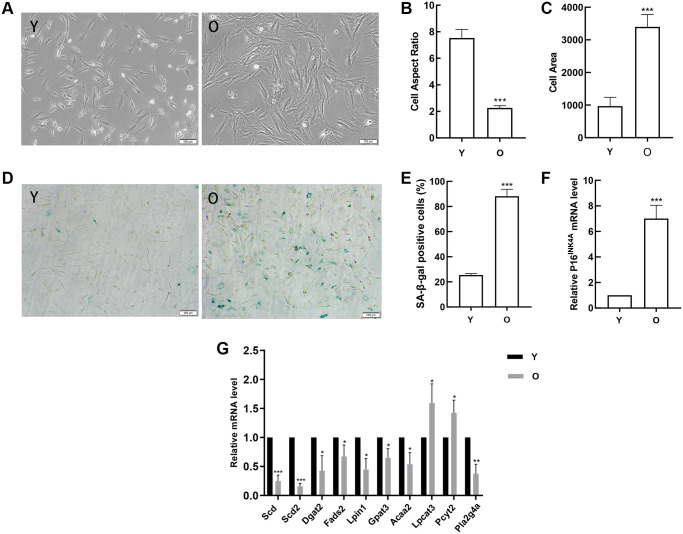
**Detection of age-related changes in BMSCs and verification of gene expression.** (**A**) Morphological alterations were observed under a phase-contrast microscope (scale bar = 100 μm). (**B**) The cell areas were obviously increased in the old BMSCs, and (**C**) the cell aspect ratios were clearly decreased. (**D**–**E**) SA-β-gal staining (scale bar = 100 μm). Senescent cells are stained blue. Compared with young cells, the proportion of positive cells in old BMSCs was significantly elevated. (**F**) RT-qPCR analyses of mRNA expression of the age-related factor pl6^INK4A^. BMSCs obtained from aged rats expressed elevated levels of pl6^INK4A^. (**G**)RT-qPCR detected the expression of the DEGs. Data indicate the mean ± SD, *n* = 3. ^*^P < 0.05, ^**^P < 0.01, ^***^P < 0.001 vs. Young (Y).

In order to verify the results of data analysis, we first obtained young and old BMSCs. With the aging of BMSCs, the cells gradually changed from long spindle to flat, lost vitality, and there were clear particles in the cytoplasm (Figure 8A). The analysis results indicated that compared with young BMSCs, the cell aspect ratios in old BMSCs decreased (Figure 8B) and the cell areas increased (Figure 8C). And the number and ratio of SA-β-gal positive cells in old BMSCs were obviously elevated by SA-β-gal staining (Figure 8D, 8E). Then the classical aging evaluation marker pl6^INK4A^ mRNA was up-regulated in old BMSCs (Figure 8F). These data above displayed that old BMSCs presented senescent alterations. Further, RT-qPCR results demonstrated that consistent with the trend of high-throughput sequencing analysis, the expressions of Scd, Scd2, Dgat2, Fads2, Lpin1, Gpat3, Acaa2 and Pla2g4a in old BMSCs were down-regulated compared with the young group, and Scd2 decreased most significantly, while the expressions of Lpcat3 and Pcyt2 were up-regulated, which was opposite to the results of high-throughput sequencing (Figure 8G).

### Scd2 over-expression ameliorates the senescence of BMSCs

**Figure 9:**
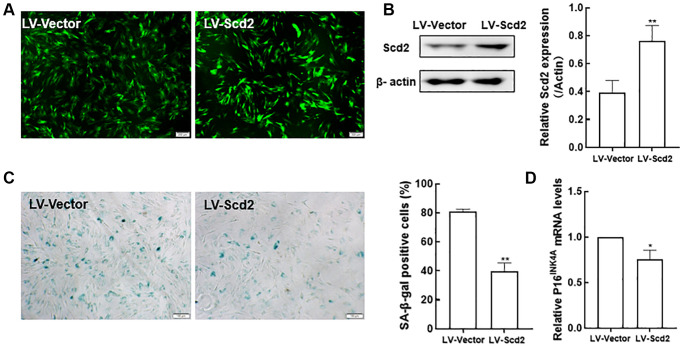
**Scd2 over-expression alleviates senescence-associated variations in BMSCs.** (**A**) EGFP expression under the fluorescence microscope (scale bar = 200 μm). (**B**) Determination of Scd2 protein expression levels by Western blot to demonstrate the transduction efficiency. (**C**) SA-β-gal staining (scale bar = 100 μm) and quantification of β-gal positive cells. (**D**) Gene expression of the senescence-related factor p16^INK4a^. Data indicate the mean ± SD, *n* = 3. ^*^P < 0.05, ^**^P < 0.01, vs. LV-Vector.

Since Scd2 gene was identified to be most significantly reduced in old BMSCs, further study was investigated whether BMSC senescence could be influenced by enforcing Scd2 expression. For this purpose, senescent BMSCs were transduced with lentivirus expressing Scd2 (LV-Scd2) and the lentiviral vector (LV-Vector). Fluorescence microscopy showed that Scd2 was successfully over-expressed in old BMSCs (Figure 9A). Scd2 expression at protein level was obviously up-regulated in the LV-Scd2 group compared with that in the LV-Vector group, which was confirmed by Western blot (Figure 9B). Furthermore, SA-β-gal staining and quantitative analysis demonstrated that SA-β-gal activity in Scd2-suffficient BMSCs was dramatically diminished in the LV-Scd2 group (Figure 9C). And P16^INK4a^ mRNA expression was markedly down-regulated after Scd2 repletion (Figure 9D). Accordingly, the above data manifested that Scd2 replenishment can attenuate BMSC senescence.

## DISCUSSION

Aging usually refers to the biological process that the functional integrity and physiological function of organism gradually decrease, leading to the decline of the ability to resist internal and external damage, and the increase of disease susceptibility and death risk [19]. With the growth of individual age, the number of MSCs reduced, and their proliferation and survival ability also weakened, which is one of the reasons for the decline of organ function in elderly individuals, and also the main factor restricting the efficacy of autologous stem cell transplantation in elderly patients [20]. Thus, we characterized the metabolomic and transcriptomic changes of BMSCs from young and old rats in a parallel and integrated way, clarified the comprehensive molecular mechanisms of BMSC senescence, and provided novel ideas and targets for the prevention and therapy of age-related diseases.

Through non-targeted metabolomics analysis, the changes of 23 different metabolites such as eriodictyol, citicoline, choline, hippuric acid, PC (16:0/18:1 (9Z)), oleic acid and fructose 1-phosphoric were obvious (Figure 2). Differential metabolites such as amino acids, organic acids and fatty acids can be used as potential biomarkers of aging. Eriodictyol can resist oxidative stress and inflammation by activating Nrf2 antioxidant pathway [21]. Citicoline decreased in the old group, which can reduce the apoptosis effect of age-related Macular Degeneration (AMD) RPE cells [22]. In addition, Citicoline has been shown in numerous studies to have a beneficial effect in slowing down neuronal aging [23, 24]. And the specific mechanism for this may be that Citicoline reduces reactive oxygen species production, stabilizes cell membranes, mitigates ischemic damage, which may provide neuroprotection by eliminating inflammation via reducing free fatty acid accumulation, blocking mitochondrial membrane components of phospholipase A2 activation, and stimulating glutathione synthesis to counteract oxidative damage [25, 26]. Perhaps there is a similar mechanism in rat BMSCs that needs to be further explored. Linoleic acid, as a precursor of arachidonic acid, produces inflammatory mediators, and it also serves as a direct target of some peroxidases to produce 9- and13-hydroxy-octadecadienoic acid and down-regulates inflammatory responses [27, 28]. Consistent with previous studies, pipecolic acid content increased with aging [29]. The levels of choline, PC and PE increased in old BMSCs, possibly due to the elevation of reactive oxygen species (ROS) that caused cell membrane damage. In brief, during the aging process of BMSCs, the antioxidant capacity declined and the lipid metabolism related metabolites changed significantly.

The role of lipid metabolism in the aging process of BMSCs was further verified by transcriptome analysis. Compared with the young cells, a total of 590 DEGs (279 up-regulated and 311 down-regulated genes) were identified in the old BMSCs. Gene ontology enrichment analysis results clarified that, wound healing, response to transforming growth factor beta, stress fiber and integrin binding were mainly enriched in senescent BMSCs, while xenobiological metabolic processes, response to lipopolysaccharide and membrane region were enriched in young BMSCs, that is, these biological processes were down-regulated in senescent BMSCs. These hypo-regulated processes may be associated with slow metabolism and physiological degeneration during aging. KEGG pathway enrichment analysis showed that Wnt signaling pathway, vascular smooth muscle contract, TGF beta signaling pathway and MAPK signaling pathway were significantly enriched in senescent BMSCs, while viral protein interaction with cytokine and cytokine receptor, metabolism of xenobiology by cytochrome P450, fatty acid metabolism, biosynthesis of unsaturated fatty acids were significantly enriched in young BMSCs. It has been shown that tissue regeneration is impaired with aging and biased towards tissue fibrosis in aged rats, and these may be related to the enhanced Wnt signaling pathway in aged rats, which may be due to increased Wnt or Wnt-like molecules in the serum of aged animals [30]. It has been shown that p38MAPK can act as an anti-aging target downstream of mTORC 1 in gut stem cells, and that activation of mTORC 1 induces p53 and p16 expression in a p38MAPK-dependent manner [31]. Cancer cells can grow rapidly and survive through reprogramming of lipid metabolism [32]. Similar to stem cells, most of lipids, such as polyunsaturated fatty acids, were easily oxidized by oxygen free radicals, resulting in cellular senescence.

After integration of transcriptome and metabolite analysis, glycerophospholipid metabolism, taurine and hypotaurine metabolism, glycerolipid metabolism, drug metabolism-cytochrome P450, drug metabolism - other enzymes were altered in senescent BMSCs, which might be the targets of anti-aging. As described previously, Ginsenoside (GRb1) can remarkably attenuate the senescence induced by physiological changes through slowing down the disorder of lipid metabolism, in which phospholipid metabolism is the most significant [33]. Our work and other studies have shown that senescent cells show overall changes in lipid composition, resulting in extensive morphological alterations and remodeling of cell membranes. Glycerophospholipids and glycerolipid metabolism were involved in aging and age-related changes [17]. Besides, taurine, as one of the intracellular antioxidants, plays an important role in oxidative stress during aging [34]. And the activity of the cytochrome P450 enzyme remains unchanged in the normal aging process, and the genetic effect is more obvious than the age effect. However, in our study, we found that the drug metabolism-cytochrome P450 pathway has changed significantly, which fully indicates that its mechanism in the aging process is more complex and needs to be further explored [35].

More interestingly, the correlation analysis between genes and small molecule metabolites showed that some genes related to lipid metabolism such as Scd2, Fads2, Lpcat3, Fads1, Scd, Pcyt2, etc. were correlated with Citraconic acid, Fructose 1-phosphate, PC(16:0/16:0), PC(16:0/18:1(9Z)), Hippuric acid, Cysteic acid, Choline, cis, cis-Muconic acid metabolites had an extremely strong positive correlation; Inpp5a, Itpr1, Cds2, Agpat5, Echs1 and Hippuric acid, PC(16:0/16 :0), cis, cis-Muconic acid, and Citicoline metabolites had a very obvious negative correlation. And it was confirmed by the analysis of lipid metabolic pathway by KEGG Pathway. This suggests that some genes related to the regulation of lipid metabolism such as Scd, Lpcat3 and Agpat5 may influence the changes of energy metabolism and thus promote or delay the aging process by regulating the related lipid metabolic pathways. Considering this, we concluded that in the aging process of rat BMSCs, the changes of lipid metabolism related genes and metabolites are more obvious, and further experiments may be performed to verify the relevant pathways to delay aging. All in all, lipid metabolism is closely related to BMSC senescence, and displays obvious changes in many genes, such as Scd, Scd2, Dgat2, Fads2, Lpin1, Gpat3, Acaa2, Lpcat3, Pcyt2, Pla2g4a and so on.

Scd, Scd2, Dgat2, Fads2, Lpin1, Gpat3, Acaa2, Lpcat3, Pcyt2 and Pla2g4a have been reported to be closely associated with cancer. For example, Dgat2 can affect the progression of hepatocellular carcinoma by regulating the cell cycle, and overexpression of Lpin1 can promote the proliferation and migration of ovarian cancer cells [36]. When RT-qPCR was performed for validation, we found that Lpcat3 and Pcyt2 expression increased with aging. Although the experimental validation results were inconsistent with the histological sequencing data, the possible reason for this is that the sequencing method of gene splicing is subject to errors. An article clearly indicates that the development of atherosclerosis is closely associated with high expression of Lpcat3 [37]. The similarly senescence indicator gene SIRT 1 is a NAD+-dependent protein deacetylase and belongs to this enzyme family. Pcyt2 expression is up-regulated in liver-specific SIRT1-deficient mice [38], so we hypothesized that Lpcat3 or Pcyt2 knockdown may delay aging. The process of senescence was accompanied by changes in energy metabolism. Aging may be associated with changes in energy metabolism during MUFA and PUFA synthesis, and the correlated genes that regulate this process, Scd, Fads 1, and Fads 2 also showed significant dysregulated expression in aged mice [39], which was consistent with our sequencing results. It has been reported in the literature that overexpression of Scd in patients with diabetes and femoral head necrosis promoted osteogenic differentiation [40, 41]. The results showed that Scd2 was most significantly downregulated in senescent BMSCs, suggesting that Scd2 may play an important role in aging and age-related diseases. Scd2 is a gene that catalyzes the rate-limiting step of monounsaturated fatty acid formation, which is important for lipid synthesis during development. The monounsaturated fatty acids are required for the maintenance of normal epidermal permeability barrier function and lipid biosynthesis [42]. The construction of some biological membrane systems such as cell membrane, mitochondrial membrane and endoplasmic reticulum membrane are closely related to lipid metabolism. In this study, we found that Scd2, a gene related to lipid metabolism, was closely associated with cellular senescence and Scd2 over-expression can ameliorate BMSC senescence. The improvement of biomembrane system has been speculated. As a key regulatory gene of lipid metabolism, Scd2 may be involved in the process of alterations in the structure of biological membrane in senescent cells. Although these differential genes have been reported to be closely related to many age-associated disorders, their role and mechanism in MSC aging are rarely reported. Therefore, it is important to explore the mechanisms related to lipid metabolism and aging of BMSCs.

Our research has some limitations. First of all, we only studied young and old BMSCs. Because of the heterogeneity and limitations of samples and techniques, the metabolites analyzed may not be complete, and not all aging studies are applicable. Secondly, due to the aged rats are difficult to obtain, the sample size of our analysis is relatively small, and more samples should be included. Finally, the exploration of BMSCs should be verified in clinical practice.

In summary, we used the method of metabolomics and transcriptome analysis to study the related mechanism of aging and metabolism with the cell samples of natural aged rats. 130 kinds of differential metabolites and 590 significantly differential RNAs were detected in the BMSCs from aged rats. Among them, 23 kinds of metabolites including lipids, fatty acids and amino acids were identified by non-targeted metabolomics. The changes of metabolites caused the alterations of 16 metabolic pathways, 3 metabolic pathways were significantly altered, and 2 metabolic pathways were potentially related to BMSC senescence. Metabolomics and transcriptomics jointly revealed that the changes of genes and metabolites related to lipid metabolism were more obvious in the aging process of rat BMSCs. Moreover, our study for the first time indicates that Scd2 repletion can attenuate the senescence of BMSCs, but further investigations are needed to unravel the underlying mechanisms.

## MATERIALS AND METHODS

### Materials and reagents

Healthy male Wistar rats, SPF grade, purchased from Animal Experimental Center, Basic Medical College of Jilin University, P.R. China. Liquid chromatography-mass spectrometry (LC-MS) grade ammonium acetate, ammonium hydroxide and MeOH were purchased from Sigma-Aldrich (USA). Acetonitrile was purchased from J.T.Baker (USA). KAPA Stranded RNA-Seq Library Prep Kit and TruSeq SR Cluster Kit v3-cBot-HS were purchased from Illumina (USA). NEBNext^®^ Poly(A) mRNA Magnetic Isolation Module was purchased from NEB (USA). RiboZero Magnetic Gold Kit (Human/ Mouse/Rat) was purchased from Epicentre (USA).

### BMSC isolation and culture

Primary BMSCs, which were isolated from the bone marrow of young (1–2 months, *n* = 3) and old (15–18 months, *n* = 3) male Wistar rats (Permit Number: SYXK 2018-0001) by whole bone marrow attachment method under aseptic conditions, were plated into 10-cm culture dishes in complete medium containing 89% Dulbecco's Modified Eagle Medium with nutrient mixture F-12(DMEM-F12) (HyClone, USA) supplemented with 10% FBS (Gibco, USA) and 1% penicillin streptomycin (HyClone, USA) [43]. The young and old BMSCs were incubated at 37°C with 5% CO2 for 24 h in a humidified atmosphere, the medium was replaced by half amount. After that, the liquid was changed every 2–3 days. When reaching 80% confluence, 0.25% trypsin-EDTA (Gibco, USA) was added to digest the cells and the subculture was continued at a ratio of 1:3 BMSCs at passage 3, young BMSCs were used as the control group and old BMSCs as the experimental group for subsequent experiments.

### Sample preparation and intracellular metabolites extraction

The culture media of young and old cells were removed, and the cells were washed with pre-cooled PBS. Then a small amount of PBS was added to the petri dish, and the cells were carefully scraped and collected into the centrifuge tube. After that, centrifugation was carried out at room temperature, 1000 rpm, for 5 min. The upper liquid was removed as far as possible without residue and the lower cell was precipitated. H2O was added into the cells at 4°C and ultrasound was performed for 10 min. MeOH and CAN were added in a 1:1 ratio to extract the spare sample 200 mL, then eddy the sample for 30s, ultrasound for 10 min. To remove the protein, the sample was incubated at −20°C for 1 h, and then centrifuged at 4°C at 20,000 rpm for 15 min. The supernatant produced by centrifugation was discarded in a vacuum concentrator and evaporated to dry. ACN and H2O were added to the precipitation at the ratio of 1:1, and then the mixture was mixed, eddy for 30s, and ultrasonic for 10 min. The insoluble fragments were removed by centrifugation at 20000 rpm at 4°C for 15 min. The supernatants were transferred to HPLC vials and stored at −80°C prior to LC/MS analysis [44]. QC samples were prepared by pooling 10 μL from each sample. The extraction of QC samples was the same as sample preparation.

### LC-MS measurements

Samples were separated on an amide column, using mobile phase A consisting of water mixed with 25 mM ammonium acetate and 25 mM ammonium hydroxide and mobile phase B ACN. The injection volume was 4 μL and flow rate was 0.4 ml/min. The generic HPLC gradient was listed in Supplementary Table 5. Then, MS analysis was carried out on the Q-Exactive MS/MS in both positive and negative ion modes.

The relevant tuning parameters for the probe were set as listed: aux gas heater temperature, 400°C; sheath gas, 40; auxiliary gas, 13; spray voltage, 3.5 kV for positive mode and negative mode. The capillary temperature was at 350°C, and S-lens at 55. A DDA method was built as follows: Full scan range: 60 to 900 (m/z); resolution for MS1 and ddMS2: 70,000 and 17,500 respectively; maximum injection time for MS1 and ddMS2: 100 ms and 45 ms; automatic gain control (AGC) for MS1 and ddMS2: 3e6 and 2e5; isolation window: 1.6 m/z; normalized collision energies (NCE): 10, 17, 25 or 30, 40, 50. Build a full scan method as follows: Full scan range: 60 to 900 (m/z); resolution: 140,000; maximum injection time: 100 ms; automatic gain control (AGC): 3e6 ions. TIC plots for sequentially selected QC samples were displayed as bellow (Supplementary Figure 1).

### Metabolomics analysis

Raw files were submitted to Thermo Compound Discover 2.1, (CD), and processed with Untargeted Metabolomics workflow with minor modification to find and identify the differences between samples. The quantification and annotation results from CD were analyzed with R script. In the analysis of multivariate data, the normalized spectral data were imported into SIMCA-P software (Version 12.0, Umetrics AB, Umea, Sweden), and the importance of low-level metabolites was increased by Pareto scaling, while the noise was not significantly amplified. Unsupervised principal component analysis (PCA) was used to observe grouping trends, highlight outliers, and show clustering between groups [45].

On this basis, the relative integral of significant metabolites determined in the OPLS-DA model was used to quantitatively analyze the metabolite levels. Variations were statistically calculated by utilizing one-way analysis of variance (ANOVA) followed by Tukey’s multiple comparison test with the Bonferroni correction using the R software (Version x64 4.0.2) and Rstudio (Version x64 3.6.2). Differential metabolites were identified with statistical significances of ^*^P < 0.05.

### RNA isolation and sequencing

When the growth of young and old cells converged and fused to 80%–90%, the culture medium was abandoned and cells were washed with PBS once or twice; 1–2 ml Trizol was added to the adherent area of 10–15 cm^2^ cells. After repeated batting for several times, the visible cell layer was completely dissolved and transferred to the RNA-free EP tube. Agarose gel electrophoresis was used to detect the integrity of the total RNA in the sample, and quantitative analysis was carried out using Nano DropNd-1000. After that, RNA was inspected. Fragmented mRNA enrichment or rRNA removal; The first strand of cDNA was generated by reverse transcription, and the double strand of cDNA was synthesized by adding dUTP. Various polymerases were added to repair the ends of the double-stranded cDNA and A was added, followed by ligase Illumina specific connector; The final RNA sequencing library was obtained by PCR amplification and purification. Agilent 2100 BioAnalyzer was used for quality control, and the final quantification of the library was carried out using qPCR method.

The sequencing library of the mixed samples was deformed by Na OH to generate single stranded DNA, which was diluted into 8p M concentration and amplified *in situ* on Tru Seq SR Cluster Kit V3-C BOT-HS. The end of the generated fragment was sequenced for 150 cycles.

### Transcriptomics analysis

The software StringTie was used to compare the results to the known transcriptome, calculate the transcriptional abundance. Differentially expressed mRNAs were detected by using the Ballgown [46]. The unit of expression amount was expressed by FPKM, and the threshold value of gene or transcript expression amount was FPKM greater than or equal to 0.5. Genes or transcripts whose mean value of FPKM exceeded 0.5 in each group are considered to be expressed in the group for statistical analysis. In addition, R packages were clusterProfiler (3.16.1) and ggplot2(3.3.2) to perform Gene ontology (GO) and KEGG pathway enrichment to describe the properties of genes and gene products of various organisms, and to determine the different biological pathways involved in mRNAs [47]. The DEGs were analyzed using STRING (http://string-db.org) database and a protein-protein interaction (PPI) network was visualized using Cytoscape (version 3.6.2).

### Integration of metabolomic and transcriptomic analysis

Metabolome and transcriptome data were visualized and clarified by using the network analysis module of online analysis software Metaboanalyst 4.0 (https://www.metaboanalyst.ca/). In addition, the metabolite differences and abundant pathways from transcriptome data were able to be clearly observed and identified.

### Analysis of the expression levels of mRNAs using RT-qPCR

The reliability of high-throughput RNA sequencing data was further confirmed. The expression levels of the top eight up-regulated mRNAs were measured using real-time quantitative polymerase chain reaction (RT-qPCR) with TransStart Top Green qPCR SuperMix (TRANS, China) in a 7300 Real-Time PCR System (ABI, Vernon, CA, United States). The primer sequences used are shown in Supplementary Table 6. SYBR Green Supermix was used for RT-qPCR. The expression was determined using the threshold crossing point (Ct) as calculated by ΔΔCt.

### Detection of senescence-associated β-galactosidase (SA-β-gal) activity

The young and old BMSCs in good growth condition were detected with the Senescence β-Galactosidase Staining Kit (Beyotime Biotechnology, China). After washing with phosphate buffered saline (PBS) and fixing, the cells were stained overnight at 37°C in the staining solution containing X-Gal. The staining was observed under inverted fluorescence microscope, and the positive cell rate (number of blue stained cells/total cells × 100%) in different areas of the culture dish was calculated.

### Lentiviral transduction of BMSCs

The optimal infection conditions of senescent BMSCs were determined according to the cell density and virus titer. The cells were inoculated at a density of 1.5×10^5^ cells/well in a 6-well plate. When about 50% confluence reached, cell complete culture medium, Scd2 over-expression lentivirus and virus infection enhancement solution were added in a certain ratio. After 12 h incubation, the original medium was discarded and replaced with new cell complete culture medium. Then EGFP expression was monitored under a fluorescence microscope and transduction efficiency was determined by western blot after subsequent 48–72 h.

### Western blot analysis

After protein extraction using RIPA lysis buffer, the total protein content was determined using the BCA protein assay kit (Beyotime). Then, 30 μg of protein extracted from each sample was resolved by 10% SDS-PAGE gels and transferred by electroblotting onto PVDF membranes (Millipore, Billerica, CA, USA). The blotted membranes were incubated with 5% skim milk for 1–2 h at RT and then detected overnight at 4°C with anti-Scd2 (1:500 dilution, Santa Cruz, USA) and anti-β-actin (1:2000 dilution, Proteintech, USA) diluted in TBST. Incubation with horseradish peroxidase-conjugated anti-rabbit IgG secondary antibody (1:2000 dilution, Beyotime Biotechnology, China) was performed for 1–2 h followed by washing with TBST. Finally, protein blots were visualized using an enhanced Electro-Chemi-Luminescence detection system (Amersham Biosciences, Inc., Piscataway, NJ, USA). β-actin was used as an internal standard.

## Supplementary Materials

Supplementary Figure 1

Supplementary Table 1

Supplementary Table 2

Supplementary Table 3

Supplementary Table 4

Supplementary Tables 5 and 6
