# Supplementary material for: A comprehensive analysis of age-related metabolomics and transcriptomics reveals metabolic alterations in rat bone marrow mesenchymal stem cells
Source: Aging (Albany NY). 2022 Jan 30;14(2):1014–32. doi: 10.18632/aging.203857 (PMC8833123; doi:10.18632/aging.203857)
Supplement: Supplementary Figure 1 [file aging-14-203857-s001.pdf]

SUPPLEMENTARY FIGURE

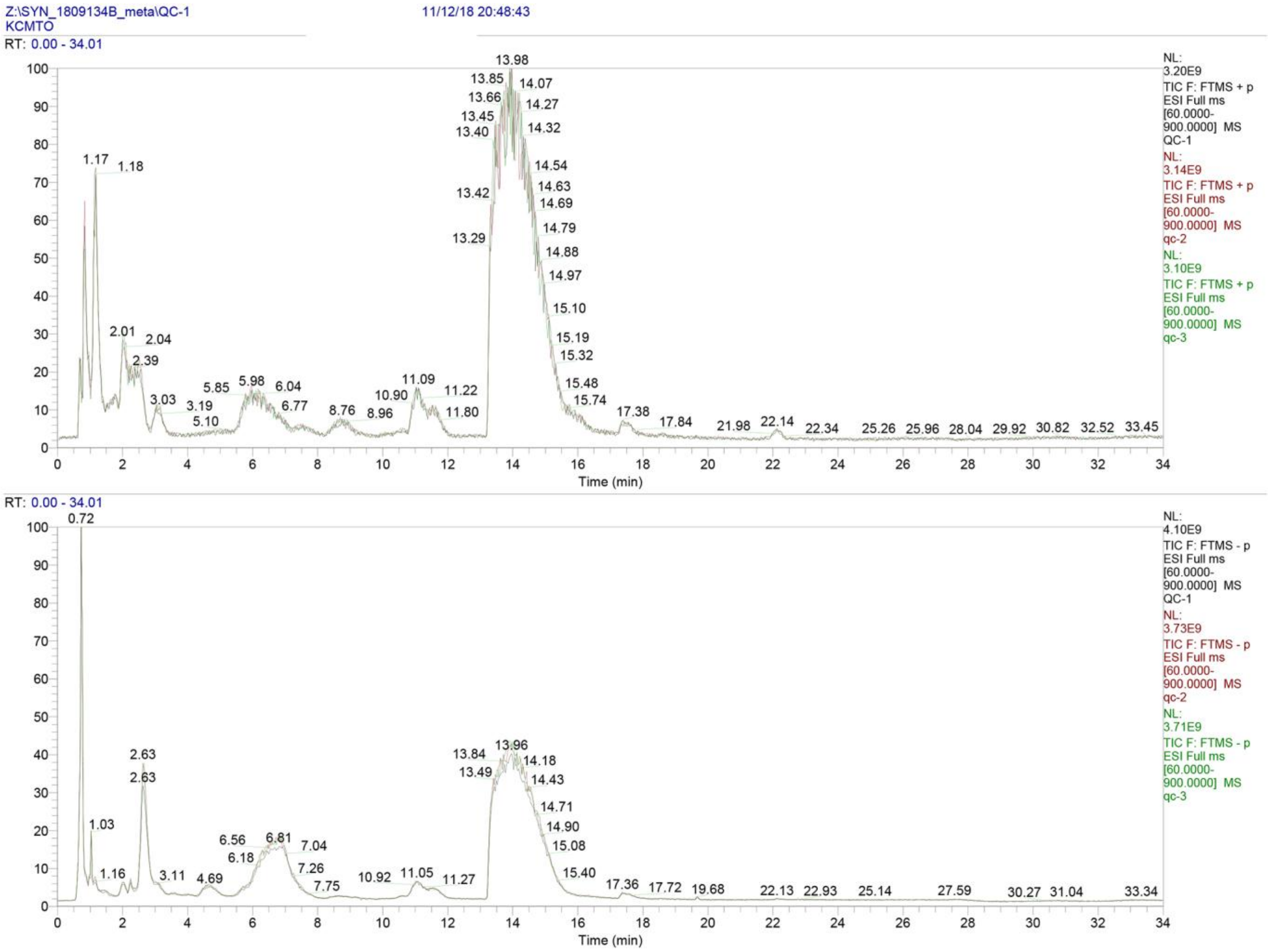

Supplementary Figure 1. TIC for sequentially selected QC samples.

Supplementary Tables

Please browse Full Text version to see the data of Supplementary Tables 1–4.

Supplementary Table 1. GO enrichment results of up-regulated DEGs.

Supplementary Table 2. GO enrichment results of down-regulated DEGs.

Supplementary Table 3. KEGG enrichment results of up-regulated DEGs.

Supplementary Table 4. KEGG enrichment results of down-regulated DEGs.

Supplementary Table 5. The generic HPLC gradient.

Time	A	B
0.0 min	10%	90%
1.0 min	10%	90%
11.0 min	13%	87%
14.0 min	20%	80%
16.5 min	30%	70%
18.5 min	50%	50%
20.5 min	80%	20%
25.0 min	80%	20%
25.1 min	10%	90%
34.0 min	10%	90%

Supplementary Table 6. Primers used for quantitative real-time PCR.

Gene	Forward primers	Reverse primers
β-Actin	5′-GGAGATTACTGCCCTGGCTCCTA	5′-GACTCATCGTACTCCTGCTTGCTG
$P16^{INK4A}$	5′-AACACTTTCGGTCGTACCC	5′-GTCCTCGCAGTTCGAATC
Scd	5′-CTCAGCGCTGGGAAAGTG	5′-GAACTGGAGATCTCTTGGAGCA
Scd2	5′-GCAGATGTTCGCCCTGAAATTA	5′-CAAATATGCAAAGAGGCAGGTGTAG
Dgat2	5′-CTTCCTGGTGCTAGGAGTGG	5′-GCCAGCCAGGTGAAGTAGAG
Fads2	5′-TTGCACAAGATTGCCCCA	5′-GGCTTCTCTTGGTATTCAATGCC
Lpin1	5′-TATGACACGGCTTGTTCC	5′-GTGGCTGCCCTGTATTTC
Gpat3	5′-TGGACTGATGGGGATCATTCAGAGA	5′-GCCCAGCTTGTCATTATCCGAA
Acaa2	5′-GCCGCCACACTATTAAGGCT	5′-CGCTTCGCAGCAACGATAAA
Lpcat3	5′-TTTCTGGTTCCGCTGCATGT	5′-CCGACAGAATGCACACTCCTTC
Pcyt2	5′-AGGCTGGGAGGTACAGAGAG	5′-AGGACATCTCCTGGCTGCTA
Pla2g4a	5′-5TGTTCAACAGAGTTTTGG	5′-AACAGAGCAACGAGATGG
