# Supplementary material for: A comprehensive analysis of age-related metabolomics and transcriptomics reveals metabolic alterations in rat bone marrow mesenchymal stem cells
Source: Aging (Albany NY). 2022 Jan 30;14(2):1014–32. doi: 10.18632/aging.203857 (PMC8833123; doi:10.18632/aging.203857)
Supplement: Supplementary Tables 5 and 6 [file aging-14-203857-s006.pdf]

SUPPLEMENTARY TABLES

Supplementary Table 5. The generic HPLC gradient.

Time	A	B
0.0 min	10%	90%
1.0 min	10%	90%
11.0 min	13%	87%
14.0 min	20%	80%
16.5 min	30%	70%
18.5 min	50%	50%
20.5 min	80%	20%
25.0 min	80%	20%
25.1 min	10%	90%
34.0 min	10%	90%

Supplementary Table 6. Primers used for quantitative real-time PCR.

Gene	Forward primers	Reverse primers
β-Actin	5′-GGAGATTACTGCCCTGGCTCCTA	5′-GACTCATCGTACTCCTGCTTGCTG
$P16^{INK4A}$	5′-AACACTTTCGGTCGTACCC	5′-GTCCTCGCAGTTCGAATC
Scd	5′-CTCAGCGCTGGGAAAGTG	5′-GAACTGGAGATCTCTTGGAGCA
Scd2	5′-GCAGATGTTCGCCCTGAAATTA	5′-CAAATATGCAAAGAGGCAGGTGTAG
Dgat2	5′-CTTCCTGGTGCTAGGAGTGG	5′-GCCAGCCAGGTGAAGTAGAG
Fads2	5′-TTGCACAAGATTGCCCCA	5′-GGCTTCTCTTGGTATTCAATGCC
Lpin1	5′-TATGACACGGCTTGTTCC	5′-GTGGCTGCCCTGTATTTC
Gpat3	5′-TGGACTGATGGGGATCATTCAGAGA	5′-GCCCAGCTTGTCATTATCCGAA
Acaa2	5′-GCCGCCACACTATTAAGGCT	5′-CGCTTCGCAGCAACGATAAA
Lpcat3	5′-TTTCTGGTTCCGCTGCATGT	5′-CCGACAGAATGCACACTCCTTC
Pcyt2	5′-AGGCTGGGAGGTACAGAGAG	5′-AGGACATCTCCTGGCTGCTA
Pla2g4a	5′-5TGTTCAACAGAGTTTTGG	5′-AACAGAGCAACGAGATGG
